# Unable to Switch Off: Fear of Missing Out, Affective Rumination, and Psychological Detachment from Work

**DOI:** 10.3390/ijerph23040463

**Published:** 2026-04-04

**Authors:** Cátia Sousa, Bárbara Pires

**Affiliations:** 1 Faculdade de Ciências Humanas e Sociais, Universidade do Algarve, 8005-139 Faro, Portugal; a71064@ualg.pt; 2 University Research Center in Psychology (CUIP), University of Algarve, 8005-139 Faro, Portugal

**Keywords:** psychological detachment, affective rumination, fear of missing out, digital connectivity, psychosocial risk, workplace health management, occupational well-being, recovery from work

## Abstract

**Highlights:**

**Public health relevance—How does this work relate to a public health issue?**

- Digital hyperconnectivity in contemporary workplaces may impair psychological detachment, a key recovery process linked to mental health and burnout prevention.
- Fear of Missing Out (FoMO) is examined as a potential emerging psychosocial risk factor that may sustain cognitive–emotional activation beyond working hours.

**Public health significance—Why is this work of significance to public health?**

- The study suggests that affective work-related rumination may represent a pathway linking FoMO to lower levels of psychological detachment.
- Findings help clarify how digitally driven psychological vulnerabilities may contribute to cumulative stress and long-term mental health risks.

**Public health implications—What are the key implications or messages for practitioners, policy makers and/or researchers in public health?**

- Workplace health policies may benefit from addressing maladaptive rumination and supporting clear digital boundary norms to protect employees’ recovery.
- Public health strategies aimed at preventing burnout may consider integrating digital-era psychosocial risks such as FoMO into occupational health frameworks.

**Abstract:**

The expansion of digital connectivity has reshaped contemporary work environments, increasing flexibility while simultaneously blurring the boundaries between work and personal life. In such contexts, employees may experience difficulties in psychologically detaching from work during off-job time. Drawing on the Effort–Recovery model and Conservation of Resources theory, this study examined whether affective work-related rumination indirectly explained the association between Fear of Missing Out (FoMO) and psychological detachment. A cross-sectional survey was conducted with 228 employees from diverse occupational sectors who completed validated measures of FoMO, affective rumination, and psychological detachment. Indirect effect analyses using bootstrapping procedures indicated that FoMO was positively associated with affective rumination, and affective rumination was negatively associated with psychological detachment. The indirect effect was significant, whereas the direct association between FoMO and detachment was not. These findings are consistent with an indirect association pattern whereby FoMO is related to lower psychological detachment through higher levels of affective rumination. However, given the cross-sectional design, the results should be interpreted as correlational evidence rather than as demonstrating a causal mediation process. The model accounted for approximately 10% of the variance in psychological detachment. Overall, the findings suggest that FoMO may be indirectly related to reduced recovery experiences via emotionally charged repetitive thinking that sustains cognitive activation beyond working hours. Addressing rumination and supporting healthier digital boundary management may therefore represent promising avenues for supporting occupational mental health in increasingly connected work environments.

## 1. Introduction

Over the past few decades, the world of work has undergone profound transformations driven by digitalization, intensification of job demands, and increasing continuous connectivity. Although these developments have enhanced organizational efficiency and flexibility, they have simultaneously contributed to the erosion of boundaries between professional and personal life. The possibility of constant contact through Information and Communication Technologies (ICTs) has made psychological detachment from work progressively more difficult, even during periods formally designated for rest [1,2]. In this context of hyperconnectivity, the ability to “switch off” has become a central challenge for contemporary occupational health.

Work recovery therefore plays a crucial role in maintaining psychological well-being. Recovery experiences refer to the processes through which employees restore their physical, emotional, and cognitive resources following periods of work-related effort [3]. Among these processes, psychological detachment—defined as mentally disengaging from work-related thoughts and concerns—has consistently been identified as one of the most critical mechanisms for preventing exhaustion, chronic stress, and burnout [4]. However, mere temporal distance from work is not sufficient to ensure effective recovery. For restoration to occur, work-related cognitive activation must be substantially reduced during off-job time.

One of the primary obstacles to this reduction in activation is work-related rumination. This phenomenon refers to repetitive and persistent thoughts about work-related issues during non-working hours, maintaining cognitive stress responses and preventing adequate restoration of personal resources [5]. Affective rumination—characterized by emotionally charged intrusive thoughts—has been associated with lower psychological detachment, poorer sleep quality, and greater emotional exhaustion. As such, it represents a key mechanism sustaining cognitive–emotional activation during off-job time.

At the same time, emerging psychosocial phenomena linked to digital hyperconnectivity may further intensify this persistent activation. Fear of Missing Out (FoMO), defined as a pervasive apprehension that one might be missing relevant experiences or opportunities, has been conceptualized as a state of heightened vigilance and continuous need for updating [6]. Within workplace contexts, FoMO may manifest as concerns about missing information, recognition, or performance-related opportunities, potentially encouraging increased attentional focus on work-related communications beyond formal working hours. This need for continued connection may increase the psychological salience of work during off-job time, fostering emotionally charged repetitive thoughts that interfere with detachment.

Despite growing attention to both FoMO and work-related rumination, research remains limited regarding the integrated examination of how digitally driven psychological vulnerabilities may contribute to impaired detachment through specific cognitive mechanisms. Understanding this dynamic is particularly relevant in contemporary work contexts, where the promotion of the “right to disconnect” and the management of employee mental health have become strategic organizational priorities.

Against this backdrop, the present study examines whether FoMO may be associated with employees’ psychological detachment through increased affective work-related rumination. By empirically testing this cognitive pathway, the present study contributes to the identification of emerging digitally driven psychosocial risks and clarifies the mechanisms through which contemporary connectivity may be related to recovery processes.

### 1.1. Work Recovery and Resource Restoration

Within increasingly digitalized and permanently connected work environments, recovery from work has become a central pillar of occupational health. Work recovery refers to the physiological, cognitive, and emotional processes through which employees restore personal resources following periods of sustained work-related effort [3]. These restorative processes are critical for maintaining psychological health and preventing cumulative strain outcomes such as chronic fatigue, emotional exhaustion, and burnout, thereby supporting sustainable functioning over time [4].

From the perspective of the Effort–Recovery model [7], work demands trigger psychophysiological activation that should naturally subside once exposure to those demands ceases. However, in contemporary contexts characterized by constant digital accessibility, this reduction in activation may be delayed or incomplete. Complementing this view, Conservation of Resources (COR) theory [8] conceptualizes recovery as a process of replenishing depleted energetic, cognitive, and emotional resources. When work-related activation persists beyond formal working hours, opportunities for resource restoration are constrained, increasing vulnerability to long-term strain.

Sonnentag and Fritz [3] conceptualized recovery experiences as four components: psychological detachment, relaxation, mastery experiences, and control. Psychological detachment refers to mentally disengaging from work-related thoughts and concerns; relaxation involves states of low physiological and emotional activation; mastery experiences entail engagement in challenging off-job activities that foster competence and growth; and control reflects perceived autonomy over how off-job time is structured. Collectively, these experiences are associated with enhanced energy levels, improved sleep quality, and reduced burnout risk [9,10].

Among these dimensions, psychological detachment has consistently been identified as a core mechanism of recovery, as it constitutes a necessary condition for the discontinuation of work-related cognitive activation [4,10]. Without mental disengagement from work-related concerns, cognitive–emotional activation remains elevated, preventing effective resource replenishment. From a resource-based perspective, failure to detach represents a sustained state of resource expenditure without adequate restoration, thereby increasing the likelihood of cumulative strain.

Recent evidence suggests that, even when employees have sufficient off-job time, many remain mentally preoccupied with work—particularly in environments marked by high digital connectivity and blurred boundaries between work and private life [4]. Understanding the psychological mechanisms that maintain the cognitive presence of work during off-job time is therefore essential. Repetitive thinking processes are especially relevant in this regard. Thus, although time away from work is necessary for recovery, it is not sufficient on its own. Successful recovery ultimately depends on the extent to which work-related cognitive activation is discontinued—a process that may be disrupted by persistent rumination. For this reason, psychological detachment was selected as the primary recovery outcome in the present study.

### 1.2. Work-Related Rumination as a Mechanism of Persistent Cognitive Activation

Rumination refers to a pattern of repetitive and persistent thinking that is difficult to disengage from, keeping individuals focused on unresolved concerns or stressors [11,12,13]. In occupational contexts, work-related rumination reflects the continuation of job-related thoughts during off-job time, thereby maintaining cognitive activation beyond formal working hours and interfering with psychological detachment [5]. Through this process, work remains psychologically salient even in periods intended for recovery.

Within the Effort–Recovery framework [7], sustained cognitive activation during off-job time represents a failure to terminate work-related psychophysiological responses. From a Conservation of Resources (COR) perspective [8], such persistent activation reflects continued resource expenditure without sufficient opportunities for replenishment. In this sense, rumination may function as a mechanism through which resource loss cycles are prolonged, increasing vulnerability to strain accumulation over time.

Research distinguishes different forms of work-related rumination that vary in content and functional implications. Affective rumination involves intrusive, emotionally charged thoughts accompanied by negative affect such as irritation, anxiety, or guilt and has consistently been identified as the most maladaptive form due to its association with sustained emotional arousal and impaired recovery [5,14]. In contrast, problem-solving pondering reflects more deliberate and solution-oriented thinking, which may occasionally serve adaptive purposes but can nonetheless prolong cognitive activation when it persists during off-job time [15]. Although multiple dimensions exist, affective rumination appears particularly detrimental because emotionally laden cognitive processing directly counteracts the disengagement required for psychological detachment.

Within the Job Demands–Resources (JD-R) framework [15], rumination may be conceptualized as a cognitive–emotional process through which job demands continue to exert strain effects beyond the workday. In this view, affective rumination represents a prolongation of demand-related activation into off-job time, thereby preventing the discontinuation of cognitive–emotional engagement necessary for resource restoration [16].

From a health perspective, the implications of this sustained activation are substantial. By maintaining cognitive and emotional engagement with work during non-working hours, affective rumination reduces opportunities for restorative experiences such as relaxation and detachment, increasing the risk of fatigue, sleep disturbances, and emotional exhaustion [3,17]. Accordingly, rumination constitutes a central pathway through which work-related demands “spill over” into private life.

Importantly, persistent cognitive activation does not occur in isolation but may be intensified by contemporary conditions characterized by continuous digital connectivity. FoMO, understood as a heightened concern about missing relevant experiences or opportunities [6], may foster vigilance and emotional preoccupation with ongoing work-related events [18,19]. From a resource-based perspective, FoMO may signal perceived threats to valued professional resources—such as information, visibility, or social standing—thereby amplifying attentional allocation to work-related cues. This heightened vigilance may promote affective rumination during off-job time, sustaining emotionally charged cognitive activation that interferes with psychological detachment.

Building on this theoretical integration, the present study conceptualizes affective work-related rumination as a potential explanatory mechanism linking FoMO to difficulties in psychological detachment. Employees experiencing higher levels of FoMO are expected to report higher levels of affective rumination, which in turn is expected to be associated with lower levels of psychological detachment during off-job time.

### 1.3. Fear of Missing Out in Digitally Connected Work Environments

FoMO has received growing scholarly attention over the past decade, particularly in the context of expanding digital connectivity. FoMO is defined as a pervasive apprehension that others may be having rewarding experiences from which one is absent, accompanied by a persistent desire to remain connected and up to date [6]. This psychological state has been associated with anxiety, heightened social comparison, and informational hypervigilance [19].

Although originally studied in relation to social media use, FoMO has increasingly been extended to organizational contexts. In the workplace, FoMO may manifest as concern about missing relevant information, professional opportunities, or signals of recognition, prompting frequent monitoring of emails, messaging platforms, and collaborative tools—even outside formal working hours [18,20]. While such behaviors may appear proactive, they may also sustain work-related cognitive activation during off-job time, thereby interfering with psychological detachment.

Recent research suggests that FoMO encompasses both internalizing and externalizing components [19]. The internalizing dimension reflects emotional experiences of insecurity and perceived exclusion, whereas the externalizing dimension captures observable checking and monitoring behaviors. In occupational settings, both components may reinforce the psychological salience of work outside working hours—either through anticipatory anxiety or through continued behavioral engagement with work-related information streams.

From a theoretical standpoint, FoMO may be conceptualized within a resource-based framework. According to Conservation of Resources theory [8], individuals strive to obtain and protect valued resources, including information, professional visibility, status, and social belonging. FoMO may signal perceived threats to these resources, increasing vigilance and attentional allocation to work-related cues. In digitally connected environments, where informational flows are continuous and evaluative signals are highly salient, FoMO may amplify sensitivity to potential losses, thereby sustaining emotional engagement with work beyond formal working hours.

Importantly, such sustained vigilance may foster affective work-related rumination. By heightening concern about missed opportunities or evaluative judgments, FoMO may increase emotionally charged repetitive thoughts during off-job time, particularly when individuals attempt to disengage from work demands. Empirical evidence has linked FoMO to difficulties in detaching from work, poorer sleep quality, and emotional exhaustion [18,21], suggesting that it may represent an emerging psychosocial risk factor in digitally connected work environments. However, the mechanisms through which FoMO may interfere with recovery remain underexplored. Specifically, the role of affective rumination as a potential mediating pathway between FoMO and impaired psychological detachment warrants further examination.

In the present study, FoMO is conceptualized as a distal psychological antecedent of affective work-related rumination. It is proposed that heightened concern about missing relevant professional experiences may increase emotionally charged repetitive thoughts during off-job time, thereby potentially reducing individuals’ ability to psychologically detach from work.

### 1.4. The Present Study

Drawing on the Effort–Recovery model [7], COR theory [8], and emerging research on digital hyperconnectivity, the present study examines whether FoMO may be associated with employees’ ability to psychologically detach from work through increased affective rumination. In digitally connected work environments, FoMO may heighten vigilance and emotional concern regarding missed opportunities or evaluative signals, thereby increasing the likelihood of emotionally charged repetitive thoughts during off-job time. These ruminative processes may prolong work-related cognitive–emotional activation beyond working hours, potentially reducing psychological detachment and, consequently, recovery.

Accordingly, this study examines an indirect effect model in which affective work-related rumination is expected to account for the association between FoMO and psychological detachment. Based on the theoretical rationale outlined above, the following hypotheses are proposed:

**H1.**  FoMO *is positively associated with affective work-related rumination.*

**H2.**  *Affective work-related rumination is negatively associated with psychological detachment.*

**H3.**  FoMO *is expected to be indirectly associated with psychological detachment through affective work-related rumination.*

## 2. Materials and Methods

### 2.1. Participants

The sample comprised 228 employed adults. Participants ranged in age from 18 to 63 years (M = 36.96, SD = 12.93), with 64.9% identifying as female and 35.1% as male. Regarding marital status, 45.6% were single, 46.1% were married or cohabiting, and 8.3% were divorced, separated, or widowed.

In terms of education, 9.6% had completed basic education, 46.5% had completed secondary education, 27.2% held a bachelor’s degree, 11.0% held a master’s degree, and 5.7% had completed postgraduate studies. Most participants worked full-time (87.7%), while 12.3% reported part-time employment. With respect to employment sector, 60.1% worked in the private sector, 36.4% in the public sector, and 3.5% in other sectors. Participants represented a wide range of occupational areas, including commerce, administrative roles, healthcare, education, hospitality, transportation, construction, and other professional domains.

### 2.2. Measures

Fear of Missing Out: *FoMO* was assessed using the Portuguese version of the Fear of Missing Out Scale (FoMOs-P) [22], adapted from the original scale developed by Przybylski et al. [6]. The instrument comprises 10 items rated on a 5-point Likert scale (1 = “Not at all true of me” to 5 = “Extremely true of me”). Although the Portuguese validation identified two correlated dimensions (internalizing and externalizing FoMO), the present study used the global FoMO score to capture the overall tendency toward fear of missing out, consistent with prior research adopting a unidimensional approach. In the current sample, internal consistency for the total scale was satisfactory (α = 0.83). Although the Portuguese validation was conducted with university students, FoMO has been conceptualized as a general psychological tendency reflecting concern about missing rewarding experiences or opportunities. In occupational contexts, this disposition may manifest as heightened vigilance regarding work-related information or professional opportunities. Therefore, the global FoMO score was used to capture the overall tendency toward fear of missing out, while acknowledging that future studies may benefit from using FoMO measures specifically developed for workplace contexts.
Work-Related Rumination: Work-related rumination was measured using the adaptation [23] of the Work-Related Rumination Scale originally developed by Cropley and Zijlstra [5]. The scale includes 15 items rated on a 5-point scale (1 = “Never” to 5 = “Always”) and assesses affective rumination, problem-solving pondering, and psychological detachment. In line with the theoretical focus of the present study, only the affective rumination subscale was used, as it reflects emotionally charged repetitive thoughts and has been identified as particularly detrimental to recovery processes. In the current sample, affective rumination demonstrated good internal consistency (α = 0.80).
Psychological Detachment: Psychological detachment was assessed using the detachment subscale of the Portuguese version of the Recovery Experience Questionnaire (REQ) [24], originally developed by Sonnentag and Fritz [3]. The full REQ comprises 16 items measuring four dimensions of recovery (relaxation, mastery, control, and psychological detachment). In line with the theoretical focus of the present study, only the psychological detachment subscale was used, as it captures the extent to which individuals mentally disengage from work during off-job time. This subscale includes 4 items rated on a 5-point Likert scale (1 = “Strongly disagree” to 5 = “Strongly agree”) (e.g., “I forget about work during my free time”). In the present sample, psychological detachment demonstrated acceptable internal consistency (α = 0.77).
Sociodemographic variables: Participants reported age (in years), gender, education level, employment status (full-time/part-time), and sector of employment (public/private). Age and gender were included as covariates in the mediation analyses because prior research has shown that demographic characteristics may influence recovery experiences and work-related rumination.

### 2.3. Procedure

Data were collected through an online questionnaire administered via Google Forms to facilitate broad access and participation. The survey link was disseminated through social media platforms, allowing for the recruitment of participants from diverse occupational backgrounds. Data collection took place between February and May 2025, and the average completion time was approximately seven minutes. Eligibility criteria required participants to be at least 18 years old and currently employed. Participation was voluntary, and no financial or material incentives were offered. Prior to participation, respondents were informed about the purpose of the study and provided informed consent. Confidentiality and anonymity were ensured, and participants were informed of their right to withdraw at any time without penalty. The study was approved by the Ethics Committee of the University of Algarve (CEUAlg) and conducted in accordance with established ethical guidelines.

### 2.4. Data Analysis

Data analyses were conducted using IBM SPSS Statistics (Version 30, IBM Corp., Armonk, NY, USA) and the PROCESS macro (Model 4, version 4.3; developed by Andrew F. Hayes) [25] to test the proposed mediation model.

Preliminary analyses included descriptive statistics (means and standard deviations) and Pearson correlations to examine bivariate associations among Fear of Missing Out, affective work-related rumination, and psychological detachment.

To test the hypothesized mediation model, a bootstrapped mediation analysis was conducted using PROCESS Model 4 [25]. *FoMO* was specified as the independent variable (X), affective work-related rumination as the mediator (M), and psychological detachment as the dependent variable (Y). Age (continuous) and gender (dummy-coded) were included as covariates in all analyses. Indirect effects were estimated using 5000 bootstrap samples and 95% bias-corrected confidence intervals. The sample size (N = 228) is generally considered adequate for mediation analyses using bootstrapping procedures, which provide robust estimates of indirect effects and do not rely on normality assumptions [25]. An indirect effect was considered statistically significant if the confidence interval did not include zero. Because all variables were collected using self-report instruments within a single questionnaire administered at a single time point, Harman’s single-factor test was conducted to assess the potential influence of common method variance. An exploratory factor analysis including all measurement items indicated that the first unrotated factor accounted for 22.71% of the total variance, which is well below the commonly suggested threshold of 50%. This result suggests that common method variance is unlikely to represent a substantial threat to the validity of the findings.

Statistical significance was set at *p* < 0.05.

## 3. Results

### 3.1. Descriptive Statistics and Correlations

Descriptive statistics and bivariate correlations are presented in Table 1. FoMO was positively associated with affective rumination, *r* = 0.23, *p* < 0.01, indicating that higher levels of FoMO were related to increased emotional rumination about work. Affective rumination was negatively associated with psychological detachment, *r* = −0.32, *p* < 0.01, suggesting that individuals who engaged more frequently in emotionally charged repetitive thoughts about work reported lower levels of mental disengagement during off-job time.

### 3.2. Mediation Analysis

To test the hypothesized mediation model, a bootstrapped mediation analysis (PROCESS Model 4; 5000 resamples) was conducted with FoMO as the independent variable, affective rumination as the mediator, and psychological detachment as the dependent variable. Age and gender were included as covariates.

As shown in Table 2, FoMO was positively associated with affective rumination, B = 0.27, SE = 0.10, *p* = 0.005. In turn, affective rumination was negatively associated with psychological detachment, B = −0.27, SE = 0.06, *p* < 0.001.

The total effect of FoMO on psychological detachment was not statistically significant, B = −0.06, SE = 0.09, *p* = 0.496. Similarly, the direct effect of FoMO on psychological detachment controlling for affective rumination was not significant, B = 0.02, SE = 0.08, *p* = 0.857.

Importantly, the indirect effect of FoMO on psychological detachment through affective rumination was statistically significant, B = −0.07, BootSE = 0.03, 95% CI [−0.15, −0.02], as the confidence interval did not include zero. This finding indicates that higher levels of FoMO were associated with reduced psychological detachment indirectly via increased affective rumination. The model explained 10% of the variance in psychological detachment (R^2^ = 0.10).

Age and gender were included as covariates in the mediation analysis. In the mediator model, both variables were associated with affective rumination, with women reporting slightly higher rumination levels (B = 0.31, *p* = 0.021) and older participants reporting slightly lower rumination (B = −0.01, *p* = 0.043). However, neither age nor gender significantly predicted psychological detachment (ps > 0.63). Importantly, the inclusion of these covariates did not alter the pattern of results observed in the mediation model.

## 4. Discussion

The present study examined the role of affective work-related rumination in the association between FoMO and psychological detachment during off-job time. Consistent with the proposed hypotheses, FoMO was positively associated with affective rumination, and affective rumination was negatively associated with psychological detachment. Importantly, the indirect effect was significant, whereas the direct effect of FoMO on detachment was not, a pattern consistent with an indirect association model. It is noteworthy that the direct association between FoMO and psychological detachment was not statistically significant. However, contemporary mediation frameworks emphasize that a significant total or direct effect is not a prerequisite for detecting meaningful indirect effects [25,26]. Bootstrapping approaches allow the estimation of indirect pathways even when the overall association between the predictor and the outcome is weak. In this sense, the observed pattern may be interpreted as an indirect association in which FoMO relates to lower psychological detachment primarily through increased affective rumination. This pattern is not uncommon in psychological research, particularly when the predictor operates through more proximal cognitive–emotional processes rather than exerting a direct influence on the outcome variable. In the present study, FoMO may represent a more distal psychological vulnerability, whereas affective rumination constitutes a proximal mechanism through which work-related cognitive activation is maintained during off-job time. From this perspective, the absence of a significant total effect does not necessarily invalidate the indirect pathway but may instead reflect the presence of multiple determinants of psychological detachment operating simultaneously. Furthermore, the magnitude of the indirect effect was relatively modest (B = −0.07), which is consistent with the multifactorial nature of recovery processes. Psychological detachment is influenced by a wide range of job characteristics, individual differences, and boundary management strategies. Consequently, single psychological variables typically account for only a limited proportion of variance in recovery outcomes. The present findings should therefore be interpreted as identifying one potential cognitive pathway linking FoMO to recovery difficulties rather than as providing a comprehensive explanation of detachment processes. Because the study relied on cross-sectional data, the indirect effect model should not be interpreted as establishing a causal sequence. Although the theoretical framework suggests that FoMO may increase affective rumination, which in turn may hinder psychological detachment, alternative temporal configurations are also plausible. For example, employees who experience persistent difficulty detaching from work may engage in greater rumination, which could subsequently amplify FoMO-related vigilance. Longitudinal and experience-sampling designs would be particularly valuable for examining these dynamic processes over time.

Although the explained variance was modest (R^2^ = 0.10), this magnitude is consistent with research examining distal psychosocial predictors of recovery processes. Psychological detachment is influenced by a wide range of factors, including job demands, job resources (e.g., autonomy and social support), and individual characteristics. Consequently, individual psychological predictors typically account for only a limited proportion of variance in recovery outcomes. Future research may therefore benefit from incorporating additional predictors known to influence recovery processes, such as job demands (e.g., workload or time pressure), job resources (e.g., autonomy, supervisory support), and boundary management strategies that regulate the interface between work and non-work domains. Individual differences related to self-regulation, technology use habits, or personality traits may also play a relevant role in shaping employees’ ability to psychologically detach from work. The present study focused specifically on the role of FoMO and affective rumination as psychological processes that may contribute to difficulties in detaching from work. Age and gender were included as covariates; however, they did not substantially alter the pattern of associations observed.

In line with recent literature extending FoMO beyond social media contexts [20,21], our findings suggest that FoMO may represent a relevant psychosocial vulnerability within contemporary workplaces where digital communication and connectivity are increasingly common. From a COR perspective [8], FoMO may reflect heightened sensitivity to potential losses of valued resources such as information, status, or professional visibility. This perceived threat can trigger sustained vigilance and emotional preoccupation with ongoing work-related events. In work environments where digital communication has become increasingly prevalent, FoMO may amplify attentional engagement with work-related cues even during non-working hours. Although such vigilance may be interpreted as proactive involvement, our findings suggest that it may carry hidden health costs by fostering affective rumination and, indirectly, reducing opportunities for effective recovery. By positioning FoMO as a distal antecedent of cognitive–emotional overactivation, the present study contributes to workplace health management literature by identifying a contemporary, technology-linked psychosocial factor that extends beyond traditional job demands.

Consistent with prior research [5,16], affective rumination emerged as a strong negative predictor of psychological detachment. Unlike more neutral forms of problem-solving pondering, affective rumination involves emotionally charged repetitive thinking, maintaining physiological and emotional activation beyond working hours. From the Effort–Recovery model perspective [7], recovery requires the cessation of work-related activation. When cognitive–emotional engagement persists, restoration of depleted resources is hindered. Furthermore, within the Job Demands–Resources (JD-R) framework [15], affective rumination may be conceptualized as a mechanism through which job demands continue to exert strain effects after work has formally ended. In this sense, rumination represents a prolongation of demand-related activation into off-job time. The indirect association pattern observed in this study reinforces this interpretation: FoMO does not directly impair psychological detachment; rather, it appears to be linked to detachment primarily through affective rumination. This finding underscores the centrality of proximal cognitive–emotional processes in understanding how broader psychosocial vulnerabilities translate into impaired recovery.

Psychological detachment has been consistently identified as the cornerstone of effective recovery [3,9,12]. While time away from work is necessary, it is not sufficient. Successful recovery ultimately depends on the discontinuation of work-related cognitive activation. Our findings align with this view by demonstrating that emotionally laden rumination significantly undermines detachment. In the context of workplace health management, this highlights the importance of addressing not only structural job demands but also internal cognitive processes that maintain work salience beyond formal working hours. By identifying an indirect association between FoMO, affective rumination, and psychological detachment, the study contributes to a process-oriented understanding of recovery failure in digitally connected environments.

### 4.1. Implications for Workplace Health Management

Although the findings should be interpreted with caution given the cross-sectional design, they offer several potential considerations for workplace health management.

First, organizations may consider FoMO as a potential psychosocial vulnerability factor in digitally intensive contexts. Policies promoting clear communication norms, reduced expectation of constant availability, and explicit support for “right to disconnect” initiatives may help reduce vigilance-driven overactivation.

Second, interventions targeting affective rumination may represent promising avenues for supporting employee recovery. Cognitive-behavioral techniques, mindfulness-based programs, and boundary management training may help employees reduce emotionally charged repetitive thinking during off-job time.

Third, workplace health management frameworks may benefit from incorporating recovery literacy, emphasizing that recovery is not merely about leisure time quantity but also about the quality of cognitive–emotional disengagement from work.

By addressing both distal vulnerabilities (e.g., FoMO) and proximal processes (e.g., rumination), organizations may be better positioned to support conditions that facilitate employees’ long-term psychological sustainability.

### 4.2. Limitations and Future Research

Several limitations warrant consideration. The cross-sectional design precludes causal inference; longitudinal or diary-based studies would allow the examination of dynamic within-person processes linking FoMO, rumination, and detachment over time. Therefore, the mediation model should be interpreted as reflecting statistical associations compatible with an indirect relationship, rather than demonstrating causal mediation. Future longitudinal and experience-sampling designs would allow researchers to test temporal precedence and within-person fluctuations. Additionally, reliance on self-report measures may introduce common method variance. Future research could incorporate objective digital trace data or physiological indicators of activation. Another limitation concerns the sampling strategy. Participants were recruited through social media using a convenience sampling approach, which may introduce self-selection bias. Individuals who are more digitally engaged may have been more likely to participate, which could be particularly relevant in a study examining FoMO and digital connectivity. As a result, the findings should be interpreted with caution regarding their generalizability to the broader working population. Because the survey was distributed through open online recruitment on social media, it was not possible to determine an accurate response rate. In addition, participants were drawn from a wide range of occupational sectors, which, although increasing ecological diversity, may also introduce contextual variability that could influence recovery processes. Moreover, the predominance of women in the sample (64.9%) may further limit the extent to which the results can be generalized to more gender-balanced working populations. Future studies would benefit from using organizational samples or probability-based recruitment strategies to strengthen external validity.

Furthermore, although this study focused on affective rumination and psychological detachment as theoretically central constructs, future research may explore whether other dimensions of rumination or recovery experiences (e.g., relaxation or control) operate differently under high FoMO conditions. Another limitation concerns the measurement of FoMO using a general FoMO scale originally validated in student samples. Although FoMO can be conceptualized as a general psychological disposition that may manifest across life domains, including work, measurement invariance between student and working populations has not yet been fully established. Future research should therefore examine the psychometric properties of FoMO instruments across different occupational samples.

Another methodological limitation relates to the use of self-report measures for all study variables collected within the same questionnaire and at a single point in time, which may increase the risk of common method variance. Although the constructs examined in this study—FoMO, affective rumination, and psychological detachment—reflect subjective psychological experiences that are typically assessed through self-report, shared measurement methods may nevertheless inflate observed associations. Future research could address this limitation by incorporating multi-method approaches, such as behavioral indicators, digital trace data, or time-lagged designs. In addition, affective rumination and psychological detachment represent closely related recovery-related constructs, which may involve some degree of conceptual and empirical overlap. Future research could further examine these constructs alongside additional recovery dimensions to better disentangle their distinct roles in work recovery processes.

In addition, although the study is framed within the broader context of digitally connected work environments, the research design did not include direct measures of digital job demands, after-hours ICT use, connectivity expectations, or remote/hybrid work conditions. Consequently, the digital work context was theoretically assumed rather than empirically measured. Future research would benefit from incorporating explicit indicators of digital connectivity at work to more precisely examine how these conditions interact with FoMO and recovery processes. Finally, as digital transformation continues to reshape work structures, examining FoMO within hybrid and remote work arrangements may provide further insight into emerging psychosocial risk configurations.

## 5. Conclusions

The present study contributes to the growing literature on workplace health management by examining the associations between FoMO, affective work-related rumination, and psychological detachment from work. The findings indicate that FoMO was not directly associated with psychological detachment; however, higher levels of FoMO were indirectly related to lower levels of psychological detachment through increased affective rumination.

These results suggest that difficulties in psychological detachment may be linked to emotionally charged repetitive thinking about work during off-job time. More broadly, the findings highlight that recovery from work depends not only on temporal distance from job demands but also on the extent to which work-related cognitive activation is reduced during leisure time.

By situating FoMO within established frameworks of occupational stress and recovery, the study offers insight into how contemporary conditions of digital connectivity may be associated with recovery processes. From an organizational perspective, the findings point to the potential relevance of interventions that support psychological detachment from work, such as initiatives aimed at reducing work-related rumination and promoting healthier digital boundary management.

Given the cross-sectional design, these findings should be interpreted as correlational evidence rather than causal relationships. Future longitudinal and experience-sampling studies would be valuable for clarifying the temporal dynamics linking FoMO, rumination, and recovery experiences in contemporary work environments.

## Figures and Tables

**Table 1:** Means, Standard Deviations, and Correlations among study variables (N = 228).

Variable	M	SD	1	2
1. FoMO	2.08	0.67	—	
2. Affective Rumination	2.52	0.99	0.23 **	—
3. Psychological Detachment	2.81	0.85	−0.07	−0.32 **

Note. ** *p* < 0.01 (two-tailed).

**Table 2:** Indirect Effect Model predicting Psychological Detachment from FoMO through Affective Rumination (N = 228).

Predictor	B	SE	t	*p*	95% CI
Mediator model: Rumination					
FoMO	0.273	0.096	2.84	0.005	[0.084, 0.463]
Gender	0.310	0.134	2.32	0.021	[0.047, 0.573]
Age	−0.010	0.005	−2.03	0.043	[−0.020, −0.0003]
Outcome model: Psychological Detachment					
Rumination	−0.273	0.058	−4.73	<0.001	[−0.387, −0.160]
FoMO (direct effect c’)	0.015	0.085	0.18	0.857	[−0.152, 0.182]
Gender	−0.056	0.117	−0.48	0.631	[−0.287, 0.174]
Age	0.000	0.004	0.10	0.923	[−0.008, 0.009]
Total effect model					
FoMO (c)	−0.059	0.087	−0.68	0.496	[−0.231, 0.112]
Indirect effect (a × b)	−0.075	0.032	—	—	[−0.146, −0.020]

Note. Unstandardized coefficients are reported. The indirect effect was estimated using 5000 bootstrap samples. Age and gender (0 = male, 1 = female) were included as covariates in all analyses. An indirect effect is significant when the 95% confidence interval does not include zero.

## Data Availability

The dataset supporting the findings of this study is publicly available on the Open Science Framework (OSF) at: https://osf.io/n7ud2/ (accessed on 3 March 2026).

## Abbreviations

The following abbreviations are used in this manuscript:

FoMO	Fear of Missing Out
COR	Conservation of Resources
JD-R	Job Demands–Resources
ICTs	Information and Communication Technologies
REQ	Recovery Experience Questionnaire
FoMOs-P	Portuguese version of the Fear of Missing Out Scale
PROCESS	PROCESS macro for SPSS
SPSS	Statistical Package for the Social Sciences
CEUAlg	Ethics Committee of the University of Algarve
